# TransFAIR study: a European multicentre experimental comparison of EHR2EDC technology to the usual manual method for eCRF data collection

**DOI:** 10.1136/bmjhci-2022-100602

**Published:** 2023-06-14

**Authors:** Nadir Ammour, Nicolas Griffon, Juliette Djadi-Prat, Gilles Chatellier, Martine Lewi, Marija Todorovic, Augustín Gómez de la Cámara, Maria Teresa García Morales, Sara Testoni, Oriana Nanni, Christoph Schindler, Mats Sundgren, Almenia Garvey, Tomothy Victor, Manon Cariou, Christel Daniel

**Affiliations:** 1 Clinical Innovation Office, Sanofi SA Recherche & Developpement, Paris, France; 2 DSI-WIND, Assistance Publique - Hopitaux de Paris, Paris, France; 3 LIMICS, INSERM U1142, Paris, France; 4 Unité de Recherche Clinique, AP-HP, Hôpital Européen Georges Pompidou, Paris, France; 5 Unité de Recherche Clinique, Assistance Publique - Hopitaux de Paris, Paris, France; 6 Université de Paris, Paris, France; 7 Janssen Pharmaceutica NV, Beerse, Belgium; 8 Instituto de Investigacion, Hospital 12 de Octubre, Madrid, Spain; 9 Biostatistics and Clinical Trials, IRCCS Istituto Romagnolo per lo Studio dei Tumori Dino Amadori, Meldola, Italy; 10 Medizinische Hochschule Hannover, Hannover, Germany; 11 Data sciences AI, Biopharmaceuticals RD, AstraZeneca FoU Göteborg, Goteborg, Sweden; 12 ICON plc, Dublin, Ireland

**Keywords:** Medical Informatics, Electronic Health Records, Data Management, Health Information Interoperability

## Abstract

**Purpose:**

Regulatory authorities including the Food and Drug Administration and the European Medicines Agency are encouraging to conduct clinical trials using routinely collected data. The aim of the TransFAIR experimental comparison was to evaluate, within real-life conditions, the ability of the Electronic Health Records to Electronic Data Capture (EHR2EDC) module to accurately transfer from EHRs to EDC systems patients’ data of clinical studies in various therapeutic areas.

**Methods:**

A prospective study including six clinical trials from three different sponsors running in three hospitals across Europe has been conducted. The same data from the six studies were collected using both traditional manual data entry and the EHR2EDC module. The outcome variable was the percentage of data accurately transferred using the EHR2EDC technology. This percentage was calculated considering all collected data and the data in four domains: demographics (DM), vital signs (VS), laboratories (LB) and concomitant medications (CM).

**Results:**

Overall, 6143 data points (39.6% of the data in the scope of the TransFAIR study and 16.9% when considering all data) were accurately transferred using the platform. LB data represented 65.4% of the data transferred; VS data, 30.8%; DM data, 0.7% and CM data, 3.1%.

**Conclusions:**

The objective of accurately transferring at least 15% of the manually entered trial datapoints using the EHR2EDC module was achieved. Collaboration and codesign by hospitals, industry, technology company, supported by the Institute of Innovation through Health Data was a success factor in accomplishing these results. Further work should focus on the harmonisation of data standards and improved interoperability to extend the scope of transferable EHR data.

**WHAT IS ALREADY KNOWN ON THIS TOPIC**

- Several articles reported on use of electronic health records (EHR) as eSource for clinical trials, however, they were performed in single centre, with a single EHR system, a single electronic data capture (EDC) system and most often not in an actual clinical study.

**WHAT THIS STUDY ADDS**

- This is the first study that proved the ability to use EHRs data as eSource in actual studies conducted by different sponsors at different sites using different EHRs systems, in different countries in Europe.

**HOW THIS STUDY MIGHT AFFECT RESEARCH, PRACTICE OR POLICY**

- The results provide practical insights to enable use of EHR2EDC technologies in actual clinical trials and help policy-makers to promote regulations to encourage adoption of EHRs as eSource in clinical trials.

## Introduction

Clinical trials complexity increased over the last decade, leading to a growing amount of data to be collected. Meantime hospitals transitioned from paper records to electronic health records (EHRs), making it possible for reuse in clinical research. Previous studies reported that 13%–75% of the trial data points are redundantly captured in EHR and the electronic data capture (EDC) system and might sometimes be present in a third paper copy.^1 2^ This results in time-consuming redundant data entry, data cleaning and source data verification, leading to an increase burden and costs.

For almost a decade, in addition to regulators, industry forums are recommending the broad implementation of EHRs as eSource in clinical trials.^3–13^ A recent literature review identified attempts to use EHR data as an eSource through direct electronic transfer into EDC systems.^14 15^ Most of the EHR-EDC integration initiatives are usually one-time-only, not scalable solutions limited to a single site, single vendor, single pharmaceutical company context, not using standards for data representation.^16–18^

Several obstacles require to be addressed to enable use of EHR data as source data in multicentric clinical trials. The main obstacles are the lack of integrated workflow between care and clinical research conducted in silos and of intersystem interoperability. Other barriers include resistance to change, and poor quality of EHR data that could influence assessment of outcomes. To improve the transparency and completeness of publications of the results of clinical trials conducted using cohorts or routinely collected data, a reporting guideline, the CONSORT-ROUTINE (extension for the reporting of randomised controlled trials conducted using cohorts and routinely collected data), has been recently developed, including a checklist to facilitate the compliance.^19^

A widely acceptable and cost-effective approach to interoperability between EHRs and clinical research systems operating under different legal frameworks across Europe^1 20 21^ was developed by the Innovative Medicines Initiative EHRs for Clinical Research (EHR4CR) project conducted between 2011 and 2016.

The EHR2EDC project, which is a continuation of EHR4CR, is a public–private partnership, funded by the European Institute of Innovation and Technology (EIT) Health involved in improving European healthcare systems. This initiative was led by Sanofi and included three other pharmaceutical companies (AstraZeneca, Janssen, UCB Pharma), a clinical research organisation (ICON), a health data technology company (InSite network platform, Custodix a TriNetX company), four European hospital organisations (Assistance Publique-Hôpitaux de Paris (AP-HP) in Paris, France; Istituto Scientifico Romagnolo per lo Studio e la Cura dei Tumori (IRST) in Meldola, Italy; Medizinische Hochschule Hannover (MHH) in Hannover, Germany and Hospital Universitario 12 de Octubre, (12 de Octubre) in Spain) and the European Institute for Innovation through Health Data (a non-for-profit organisation). The aim of this project was to design, develop and evaluate a technology enabling use of EHRs as eSource in clinical trials.^22^

The objective of the EHR2EDC consortium was to prove that at least 15% of data entered in the EDC can be semiautomatically transferred from its source EHRs. To evaluate this the TransFAIR study was designed, within relevant context of use, by including six different clinical studies across three research sites in Europe. The primary endpoint was the ability to achieve 15% of correct and accurate data transfer from EHRs to study EDC. This percentage was agreed as a consensus, and based on published work on this subject, such as the RE-USE project.^1^

## Methods

### Study design

The TransFAIR study consisted in the experimental comparison of two data collection methods: the EHR2EDC module implementing a semiautomatic transfer of EHR data to an EDC system versus the usual manual data collection (protocol available in online supplemental material). We included real ongoing clinical trials (support CT). Selected trials were conducted according to their protocol and were not affected by the TransFAIR study. FAIR refers to the FAIR principles: Findability, Accessibility, Interoperability and Reuse of data assets guided the design of the EHR2EDC module.^23^

10.1136/bmjhci-2022-100602.supp1 Supplementary data

Data were shared between partners according to the European Union General Data Protection Regulation. The interoperability implementation and data flow were performed within a solution compliant with data privacy and good clinical practice regulations.

### EHR2EDC data in scope, module setup, study and patient selection

The data domains of interest were selected based on the frequency of data types collected in a large pool of studies (N=120) and present across multiple therapeutic areas. The results were reviewed by members of the project experts in clinical data standards with extensive experience in designing study eCRFs, including experts from the clinical research organisation (CRO) ICON, for their experience across sponsors and therapeutic areas. The four data domains selected are: demographics (DM), vital signs (VS), laboratory (LB) and concomitant medication (CM), from where a core set of 48 Clinical Data Interchange Standards Consortium (CDISC) data elements was identified and the 20 associated CDISC code lists were mapped to selected terminologies (International Classification of Diseases, 10th Revision (ICD-10), Logical Observation Identifiers Names and Codes (LOINC), Anatomical Therapeutic Chemical (ATC) Classification and Systematised Nomenclature of Medicine Clinical Terms (SNOMED-CT)). CDISC standard is the destination format selected as it is used by pharmaceutical companies or CRO for their eCRF. The semantic mappings developed for this project is accessible at the following site:

It covers four CDISC domains: DM, LB analysis, VS and CM. LOINC is the main reference terminology used on hospital side, however, it has sometimes been necessary to use other terminologies.

Four Fast Healthcare Interoperability Resources (FHIR) profiles associated with a list of standardised value sets were defined to support data extraction specification and guide mappings done by hospitals terminology experts.

The EHR2EDC module, from the InSite platform has been installed successfully in: AP-HP, 12 de Octubre, IRST and MHH.

Six studies from three different Sponsors (AstraZeneca, Janssen and Sanofi) were selected by the consortium according to the following criteria: support CT had to be conducted in a hospital partner with principal investigators agreeing to support the TransFAIR study, it had to include patients during the evaluation period (July to December 2019) and preferably collecting a large number of LB data.

The selected studies were conducted in three hospitals: AP-HP, 12 de Octubre and IRST. MHH only started to map on SNOMED-CT, with weekly data refresh from the clinical live systems, hence was not included.

### Data collection and management

**Figure 1:**
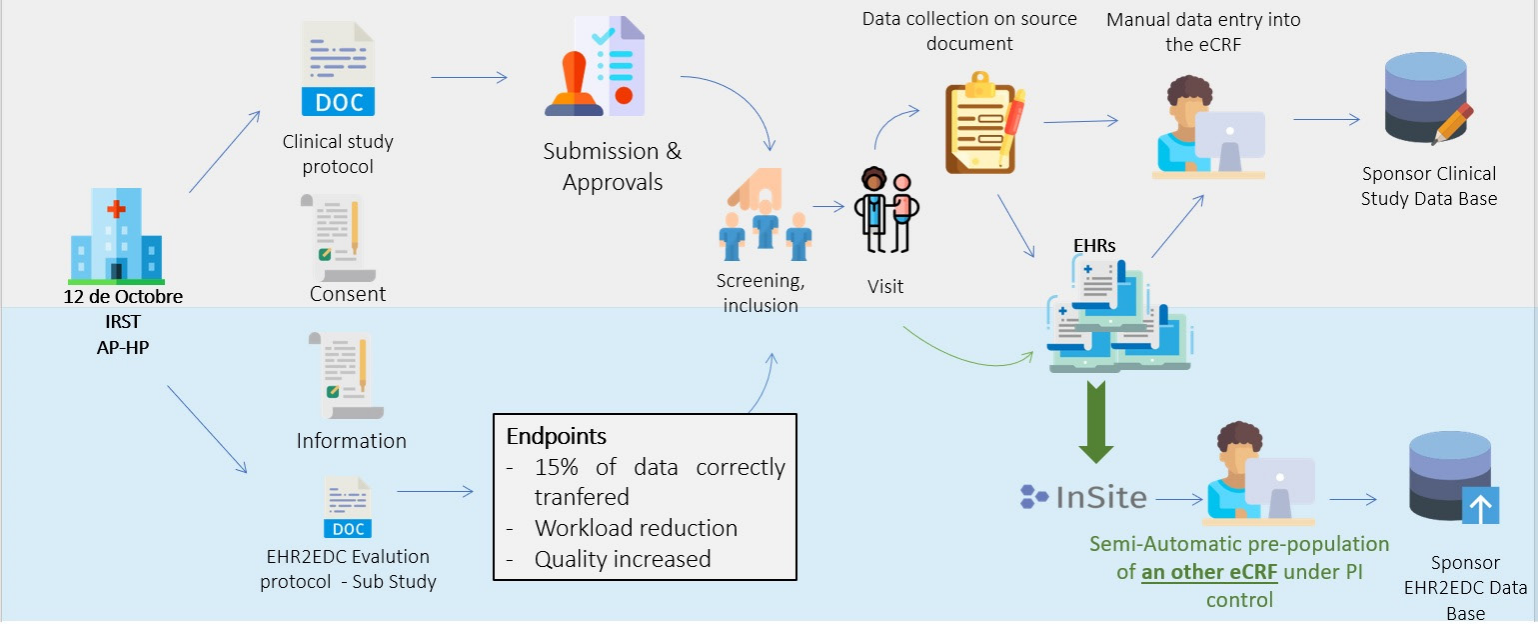
General organisation of the TransFAIR study. AP-HP, Assistance Publique-Hôpitaux de Paris; EHR2EDC, Electronic Health Records to Electronic Data Capture; PI, principal investigator; eCRF, electronic Case Report Form.

**Figure 2:**
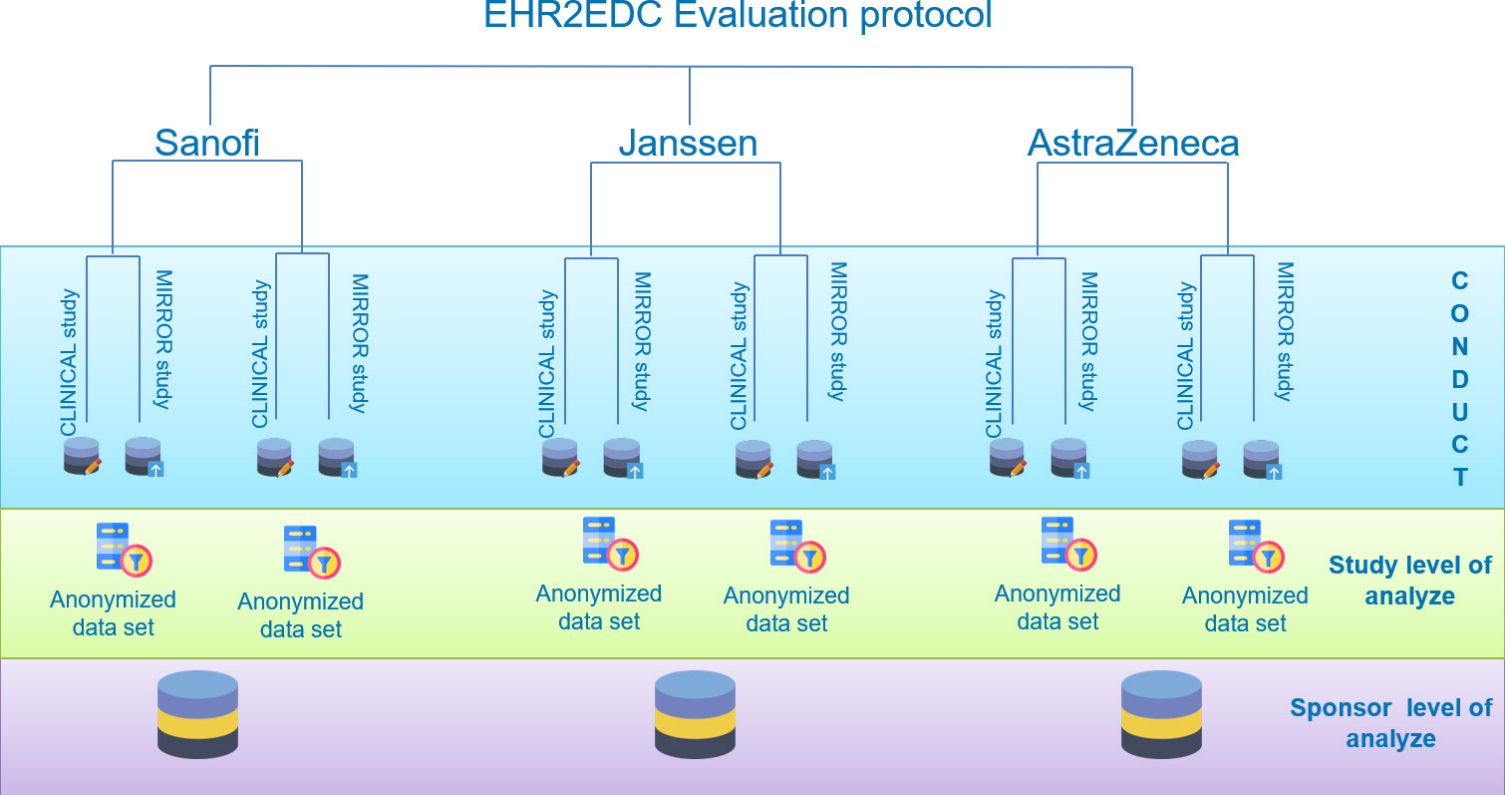
Mirror study representation. EHR2EDC, Electronic Health Records to Electronic Data Capture.

For each clinical trial selected for the TransFAIR study, a mirrored EDC database, replicating the study specific EDC database, was set up and connected to the EHR2EDC module of the InSite platform installed at each site. The mirror EDC database represents the ‘experimental’ database while the original database was used as a ‘control’ (figure 1). The data collected in each EDC system of participating clinical trials were captured in the study eCRF (Medidata Classic Rave V.2020.2.0) using traditional manual data entry by a study coordinator or an investigator. In the mirrored database, the same data were collected through the InSite platform (figure 2). Once connected to the InSite platform data, the study coordinator/investigator selects a clinical trial, a subject and a visit (as defined in the protocol). Then he/she must associate the visit to the actual date of the patient’s visit. The platform provides an interface, with fields prefilled with EHR data (required by study protocol) at the selected date. The study coordinator/investigator can, therefore, review and validate data before their transfer to the mirror EDC.

Several patients were included, and visits completed before the TransFAIR study started. Data were transferred, retrospectively for completed visits and for new visits. Investigators supervised the automated data collection by reviewing, validating and transferring data to the experimental database. Experimental and control databases were then reconciled by the sponsor to identify discrepancies. An absence of difference between data points collected in both databases was classified as OK, while a difference was classified as NOK. Each discrepancy was investigated by the investigator by checking source documents to verify the actual value of the data point for which a discrepancy was identified and to document the reason.

### Study endpoints

The primary endpoints are the percentage of data points accurately processed.

- Per individual studies.
- Across studies.

The secondary endpoints are the percentage of data points in scope accurately processed.

- Per individual study.
- Pooled across studies.
- Per data domain pooled across studies.

### Statistical analysis

Since the TransFAIR was a proof-of-concept study, neither a sample size calculation nor a power consideration was performed. The only hypothesis to be tested was that at least 15% of the datapoints could be semiautomatically and accurately transferred by the EHR2EDC module. Results were analysed individually, for each study and pooled together to be presented across studies.

The percentage of data accurately transferred was calculated as the number of data correctly transferred in the experimental database divided by the total number of data manually entered into the control database.

The hypothesis of transferring at least 15% of the data was tested using a one-sided exact binomial test. An estimate of proportions with their 95% CI was provided. The exact calculation method was used if the approximation of the Normal law was not possible. Subgroup analyses were planned on the following variables: study site and data domain (DM, VS, LB and CM).

The statistical significance level was set at p<0.05 (two sided). The global statistical analysis was carried out with the R software (release V.3.6.3; R Foundation for Statistical Computing, Vienna, Austria), by the Clinical Trial Unit of each site and by ICON.

## Results

### Presentation of the studies and patient data

The EHR2EDC transfer module of the InSite platform was active from 20 September 2019 to 30 November 2019. The analysis included the data points of five of the six selected studies: AZ D169CC, PCR3001 and TED14856 at 12 de Octubre in Madrid, BCL30003 and D19BC at IRST. The data from the EFC14875 study at AP-HP were excluded from the overall analysis. Most data collected for that study, at that site, were captured using paper as a source.

**Figure 3:**
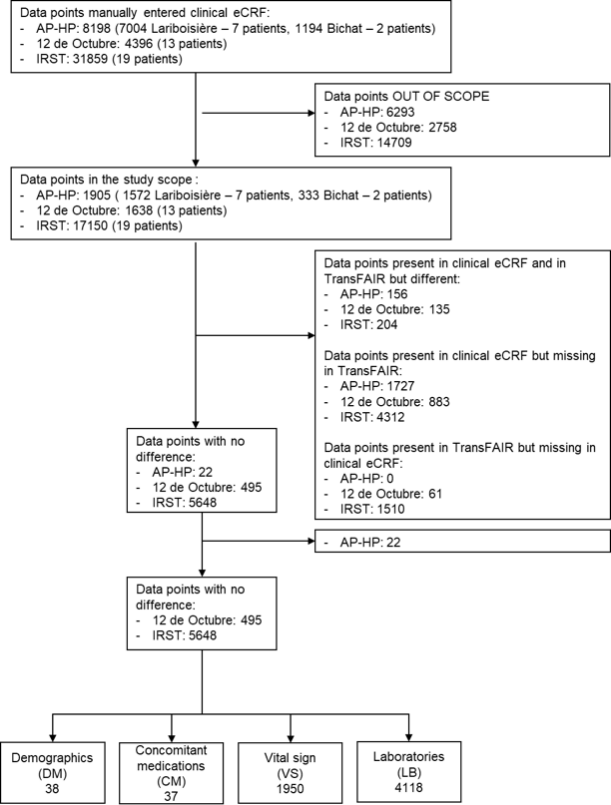
Study flow chart. AP-HP, Istituto Scientifico Romagnolo per lo Studio e la Cura dei Tumori; IRST, Istituto Scientifico Romagnolo per lo Studio e la Cura dei Tumori; eCRF, electronic Case Report Form.

The data from the five studies databases were pooled and represented a total of 41 424 data points. The subset of data in the scope of the study (ie, DM, VS, LB and CM) represented 19 240 data points, 46.4% of total data collected (figure 3).

### Primary endpoint: percentage of data accurately transferred (all data)

- Per individual studies

Studies TED14856 and AZ D19BC had reached higher results than set objective of 15%. They achieved, respectively, 26.5% (one-sided 95% CI 24.0%) and 22.8% (one-sided 95% CI 22.2%) (table 1).

**Table 1:** Percentage of accurately transferred data, overall and by study

Hospital and study	No of data accurately transferred (n)	% of accurately transferred data	
		In the scope of the TransFAIR study % (95% CI lower limit)	For the whole study % (95% CI lower limit)
12 de Octubre	495	32.7% (30.3%)	11.3% (10.3%)
AZ D169CC (AstraZeneca, NCT03619213)	143	26.2.% (22.4%)	7.8% (6.6%)
PCR3001 (Janssen, NCT02257736)	35	25.6% (18.5%)	2.6% (1.8%)
TED14856 (Sanofi, NCT03284957)	317	35.0% (31.9%)	26.5% (24.0%)
IRST	5648	55.6% (54.6%)	17.7% (17.3%)
BCL30003 (Janssen, NCT03390504)	400	60.3% (56.5%);	6.7% (6.0%)
AZ D19BC (AstraZeneca, NCT02516241)	5248	55.2% (52.4%)	22.8% (22.2%)
Total	6143	39.6%	16.9% (16.6%)

IRST, Istituto Scientifico Romagnolo per lo Studio e la Cura dei Tumori.

Other studies achieved less than 10.0% of correctly processed data.

- Across studies

The EHR2EDC module was able to transfer accurately 16.9% of data points across studies, (one-sided 95% CI 16.6%) and represents 6143 data points.

### Secondary endpoints (data in scope)

- Results per individual study varies between 26.6% and 60.3% (table 1).

The AZ D19BC trials and TED14856 trial both had a majority of VS and LB data (table 2).

**Table 2:** Data transferred per domain and per study

Hospitals and studies	Patients (N)	Break down of data points by data domain and by study				
		CM	DM	VS	LB	Total
12 de Octubre	13	385	90	564	599	1638
AZ D169CC (AstraZeneca, NCT03619213)	8	126	28	394	0	548
PCR3001 (Janssen, NCT02257736)	4	72	52	61	0	185
TED14856 (Sanofi, NCT03284957)	1	187	10	144	564	905
IRST	19	156	41	4541	12 412	17 150
BCL30003 (Janssen, NCT03390504)	2	156	41	40	449	686
AZ D19BC (AstraZeneca, NCT02516241)	17	0	0	4501	11 963	16 464

CM, concomitant medications; DM, demographics; IRST, Istituto Scientifico Romagnolo per lo Studio e la Cura dei Tumori; LB, laboratories; VS, vital signs.

- Results pooled across studies: The EHR2EDC module was able to process accurately 39.6% (p<0.0001) of data points in scope (N=6143 data points) (table 3).
- Results per data domain pooled across studies: Within each data domain in scope, the percentage of data correctly processed varies. The highest results are observed for VS (40.9%), LB (40.6%) and for DM (34.2%). Data from CM have the lowest percentage: 7.7% (table 4).

**Table 3:** Number of data points per data domain

N (%)	DM	CM	VS	LB	Total
No difference	38 (0.2)	37 (0.2)	1950 (12.6)	4118 (26.6)	6143 (39.6)
Missing in TransFAIR	73 (0.5)	432 (2.8)	2566 (16.6)	4373 (28. 2)	7444 (48.0)
Different	0 (0.0)	4 (0.0)	150 (0.1)	185 (1.2)	339 (2.2)
Missing in clinical eCRF*	0 (0.0)	10 (0.1)	104 (0.7)	1457 (9.4)	1571 (10.1)
Total	111 (0.7)	483 (3.1)	4770 (30.8)	10 133 (65.4)	15 497 (100.0)

eCRF, electronic Case Report Form.

*Excluded from total.

CM, concomitant medications; DM, demographics; LB, laboratories; VS, vital signs.

**Table 4:** Proportion of data collected and not collected for the four domains in the TransFAIR study scope

Data domain	% of data correctly transferred	% of missing data
Demographics	34.2	65.8
Laboratories	40.6	59.4
Vital signs	40.9	59.1
Concomitant medications	7.7	92.3

## Discussion

The concept of mirror study has proven to be an effective method for validation of a novel technology to support data collection, in a relevant context of use: different EHRs, investigation sites, sponsors and studies.

The primary objective of the study was successfully met, with over 15% (16.9%) of the data points entered in the e-CRF correctly processed from EHR source records.

The four domains DM, VS, LB and CM selected by the consortium represent 46.4% of the data collected through the five trials in scope, this results validates the consortium choice.

A per study analysis demonstrates the major contribution of the local LB data followed to a lesser degree by the VS data to achieve an acceptable proportion of transferable data. This suggests that studies in oncology (ex: TED14856 and the AZ D19BC), with high volume of local LB data are best candidates for the early use of this digital data collection technology in the near future.^24^

The two domains LB and VS covers around 40% of the data in scope and represent more than 96% of accurately transferred data. This reflects the availability and good quality of these data at the hospitals EHRs.

The interoperability challenge has been successfully addressed through the implementation within the EHR2EDC module of a core list of data elements and its associated library of terminology mappings. The EHR2EDC module has been efficiently deployed in the four hospitals and the different users trained. The mapping and its implementation were designed to be reusable across studies, with limited (re)verification activities, to provide operational efficiencies, both for the sponsor and for site staff.

The limitations on the results for data in scope highlight a combination of factors affecting the ability to achieve higher performance. Among those factors, we have identified several root causes with possible remediations:

### Regulations

For DM data (DM domain), legal limitations in collecting ethnicity in Europe produces an artefact as this information is collected during trials. When analysing only legally acceptable DM data, the result was 100%. This suggests that calculation methods and possible automatic quality controls must consider local regulations to be accurate.

### Case report form design

The primary cause of missing data for the VS and LB domains arises for specific data points collected in study eCRFs to document the execution of the procedure. Most of the empty fields expect a ‘yes’ value for the question ‘Has the test been performed?’/‘Was the blood sample taken?’. This could be resolved by using auto populated fields (updated to a ‘yes’ value if results are present).

### Local investigator’s team practices

Unlike IRST, other hospitals did not routinely train their staff to fill-in structured forms of the EHRs, and so the proportion of data accurately transferred was adversely affected by the proportion of data collected in EHR as free text or in paper source documents when running a clinical trial.

Special attention should be focused on staff using EHRs to collect patient data associated with a clinical study for preventing free text data entry or paper source. This includes training hospital staff in data quality standards, upgrading quality assurance measures and strengthening data governance activities, to enable EHR data to be trustworthy reused in research.

In the TransFAIR study, the low percentage of CM data correctly transferred reflects that they are more often recorded as free text, for example, in unstructured documents (eg, doctor’s letters) and a large part is prescribed outside of the investigational site and is consequently not captured in the EHR.

### Clinical site maturity/readiness

Other factors influencing the level of performance include the site maturity in using their EHRs for clinical trials activities. Site organisational capabilities, best practices (EHR data quality assurance, use of EHRs as eSource in clinical trials, just-in-time data flow), skilled staff (data integration, data management) are essential to benefit from this new method of digital data collection.

Guided work effort is needed to augment the proportion of data recorded as eSource in EHRs to be collected using EHR2EDC solutions. Initial focus would expand transferability of structured data in EHRs, and work at rendering unstructured data to be collected. We envision this effort to be made possible through the development of consensus on ‘high-value data sets’, representing the data most commonly collected in clinical trials.

Nevertheless, not all data collected in clinical trials has its correspondence in patients’ EHRs sources. For example, specific forms in eCRFs collect data in relation with the management and evaluation of investigational medicinal products (tracking, patient’s compliance, pharmacokinetic data, etc).

## Conclusion

Overall, a 16.9% successful transfer rate was achieved across the five trials included in the TransFAIR study. A transfer rate of 26.5% of data used as eSource EHRs was achieved in one of the trials.

Clinical investigational sites, CRO staff and sponsor personnel involved in the planning and the execution of trials, as well as those involved in the management of EHR, EDC and EHR2EDC technologies must join forces for success. It is recommended to promote coordination and synchronisation of all actors to align, not only on the European EHR technology standards, but also on addressing the following different dimensions: change management, and new roles, needed to achieve routine use of EHR data as eSource in clinical trials.

A roadmap to transition use EHR2EDC in clinical trials would include the following recommendations: (1) Sponsors should further develop sets of high value data, combining structured and unstructured data to help guide and prioritise the efforts needed for scalability. (2) Clinical sites should initially focus on structured data, such as LB, DM, VS and CM using common data models, for example, HL7 FHIR, increasingly implemented in clinical research^25–27^ and reference terminologies for example, ICD10, LOINC, ATC, SNOMED, etc. (3) Clinical sites should develop capabilities to leverage data from unstructured format (free text, clinical documents, images), not standardised data, using natural language processing technologies and efforts to enhance both data interoperability and data quality controls. and (4) Collaborative effort at the ecosystem level should be encouraged to create the right incentives to develop and grow the market with technology providers to offer EHR2EDC services to sponsors’ organisations.

## Data Availability

No data are available. Data are not available as sharing the data as public acess was not part of the inform consent signed by patients.
