# Supplementary material for: TransFAIR study: a European multicentre experimental comparison of EHR2EDC technology to the usual manual method for eCRF data collection
Source: BMJ Health Care Inform. 2023 Jun 14;30(1):e100602. doi: 10.1136/bmjhci-2022-100602 (PMC10277109; doi:10.1136/bmjhci-2022-100602)
Supplement: Supplementary data [file bmjhci-2022-100602supp001.pdf]

PROTOCOL SUMMARY

SYNOPSIS

Protocol title: Use of Electronic Health Records as eSource; Multicenter European study, comparing manual data collection in an eCRF to data collection using EHR2EDC technology

This is a prospective European comparison of a new computer tool for data collection versus manual entry.

Short title: TransFAIR Study

Protocol Summary: This is a prospective comparison of a new computer tool for data collection versus manual entry.

For this evaluation, several Clinical Trials (CTs) sponsored by EFPIA Partners (Astra Zeneca, Janssen and Sanofi) have been identified as planned or in progress in partner hospitals of the EHR2EDC project. This includes AP-HP (France), 12 Octubre (Spain), IRST (Italy) and MHH (Germany).

These CTs will be conducted in a completely usual way, and will not be affected by the TransFAIR study. The data collected in the CSs will serve as a control. They are named "**CONTROL Data**”.

On a top of each CT, the EHR2EDC autocomplete module will be implemented in a separate mirror study to be used as the experimental arm. It will collect the "**TransFAIR Data**". The data collected in this way will be compared to the CONTROL Data of its sister CT, to evaluate the new tool.

Figure 1; TransFAIR Study

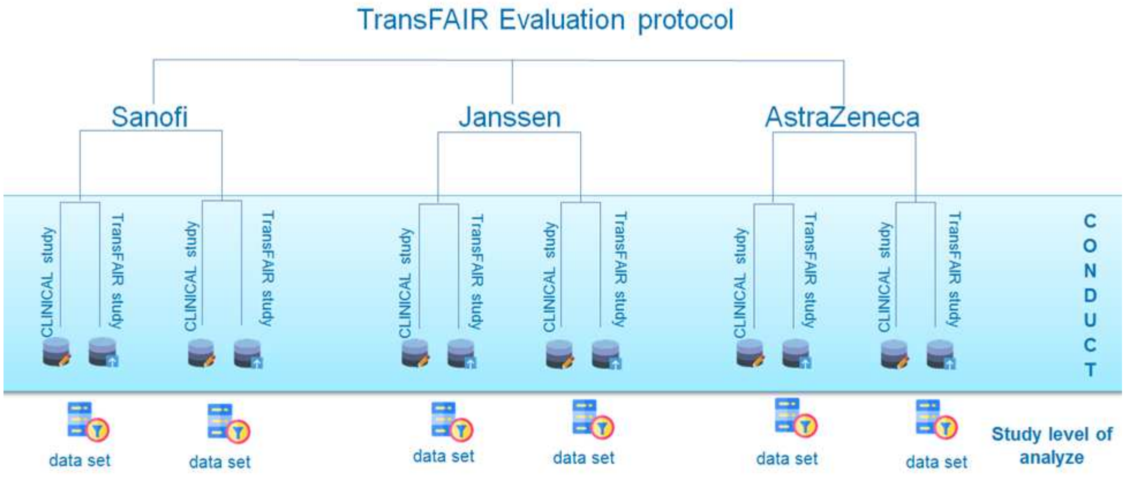

The TransFAIR study is not a clinical trial, it is a technology evaluation study. The data collected in the TransFAIR study must not be used for any regulatory purpose. Only data collected in the traditional clinical trials (Control) are eligible to support regulatory obligations.

Ammour N, *et al. BMJ Health Care Inform* 2023; 30:e100602. doi: 10.1136/bmjhci-2022-100602

Patients included in the traditional clinical trials, will be informed that their data, collected in the context of the traditional clinical trials (Control) are expected to be collected in the TransFAIR technology study, after receiving their verbal or written consent.

Figure 2 : Clinical Study vs. TransFAIR study Design
Vv

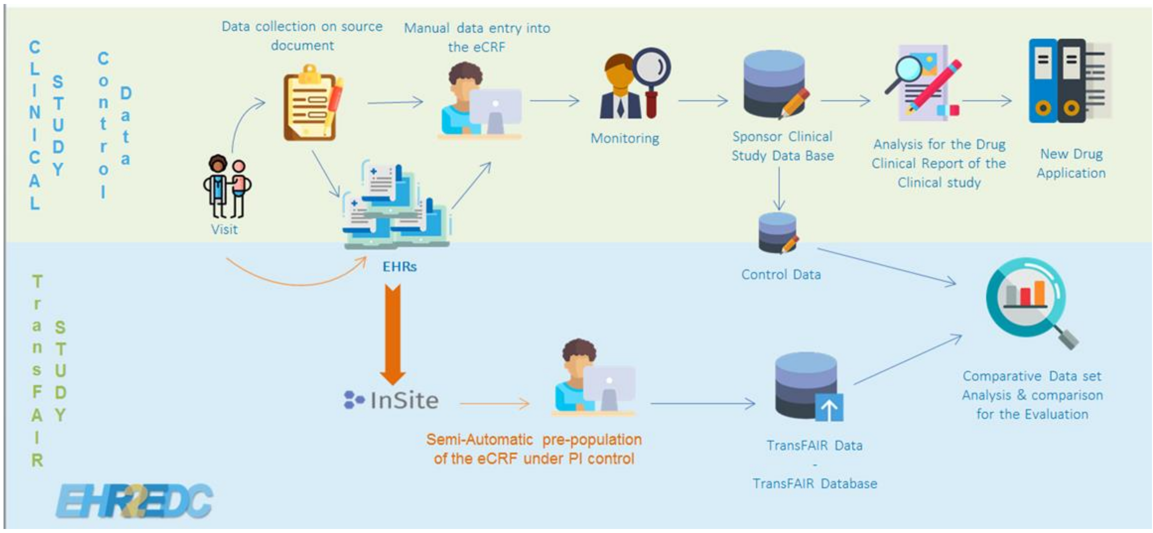

For the purpose of the study, each protocol in in the TransFAIR study will have a specific data base setup in mirror of its Clinical Trial sister, this way nullifying the impact on the CT.

Context

The Center for Drug Evaluation and Research (CDER) encourages seamless exchange of structured, re-usable information between health care and clinical research systems so that data may be entered once at the point of care and used many times without manual re-entry or manual source data verification. In September 2013, FDA published the Electronic Source Data in Clinical Investigations guidance promoting the need for capturing source data in electronic form including data originating from health care systems. Furthermore the FDA has release a new guidance in July 2018 - Use of Electronic Health Record Data in Clinical Investigations. This guidance is intended to assist sponsors, clinical investigators, contract research organizations, institutional review boards (IRBs), and other interested parties on the use of electronic health record data in FDA-regulated clinical investigations.
The value of collecting data directly from EHRs to sponsor EDCs is widely recognized, and offers tremendous promises to streamline medical research, accelerate development of new cure and deliver treatments innovations to patients sooner and cheaper. However, disparate siloed healthcare systems, coupled with complex regulatory framework makes it very difficult today to leverage the data asset in a data privacy and ethically proven way.

The EHR2EDC consortium has established as an objective to address the barriers and challenges to use patient EHRs data as an acceptable eSource and enable seamless data transfer of patient EHRs data to sponsor EDCs system for analysis and generation of evidences. Beside using data for clinical

Ammour N, *et al. BMJ Health Care Inform* 2023; 30:e100602. doi: 10.1136/bmjhci-2022-100602

investigations of new drugs, this will be a key infrastructure component of the health learning system approach aiming at using health data to improve provision of care to patients.

The EHR2EDC consortium has developed a generalizable module allowing semi-automatic transfer (under control of the principal investigator and delegated personnel) of patient data and provenance metadata from Electronic Health Records (EHRs) to Electronic Data Capture systems (EDCs), (i.e. eCRFs). The purpose of this study is to evaluate capability of the EHR2EDC module in real life with real patient data via several clinical protocols in different therapeutic areas, conducted at several investigational sites across Europe.

Evaluation plan and methods

This study deals with a technological evaluation, with the objective of semi-automatically* processing a certain number of data usually collected and transcribed manually. The unit of analysis is therefore the data element as entered in an eCRF. The data collected relates to the patients included in the study period transFAIR (or earlier) in one of the trials listed in Table 1 (List of Candidate reference Clinical trials).

The data included in this evaluation are the data for which the EHR2EDC project produced and delivered a mapping catalog to the EHR2EDC pivot model.

The data domains concerned:
- Laboratory examinations
- Demographics
- Vital signs
- Procedures,
- Diagnostic
- Prescriptions (before, during and during hospitalization)
- Present and past medical conditions
- Other (to be further defined, ex:

The control data will firstly be collected and monitored by the sponsor monitors (queries and resolution) in the eCRFs of the reference studies (Control Data). After collection in clinical trial eCRFs. The same data will be collected in the TransFAIR study using the EHR2EDC device.

This evaluation will demonstrate the added value of such technology in clinical trials for hospital site staff in terms of the reduction of time and effort for data collection and entry, and an increase of the data quality of the EDC.

**The term "semi-automatic" is equivalent to "automatically transferred under control of Principal Investigator and delegated personnel".*

Objectives and end points

Objectives	Endpoints
**Primary**	
To assess the percentage of data that can be processed by EHR2EDC	To demonstrate at least 15% of the data usually manually entered into the eCRF can be semi-automatically and accurately transferred into the EDC using EHR2EDC module.
**Secondary**	**Measure**
To compare EHR2EDC data collection steps and activities versus the manual data entry process at site level	For each actor the steps and activities will be described and documented for each separate process, to compare the number of steps needed for each process.
To compare EHR2EDC data collection workload versus the manual entry process at the site level	For each actor the workload will be documented through surveys (& system/self-reporting) to assess time, effort and usability of each process.
To compare data management activities with or without the EHR2EDC solution the sponsor level	For each study the number of queries (data related enquiries) generated will be compared for each process (feasibility to be confirmed)
To compare the data accuracy of the EHR2EDC databases versus CTs databases.	Erroneous data in the two database with the same dataset.
**Exploratory**	
To assess the generalizability of the EHR2EDC module	Description and characterization of :<br>- sponsor and site workload between studies from different sponsor<br>- data correctly transferred (percentage of the total data transferred and classification of data by domain)
To qualify the data quality of each database by comparing the data queries of both databases	Characterization, classification and quantification, of the data manager queries and compare the data queries of both databases

Ammour N, *et al. BMJ Health Care Inform* 2023; 30:e100602. doi: 10.1136/bmjhci-2022-100602

Two periods must to be distinguished:

- Retrospective period: will focus on a transfer of data already collected during the RCTs to be semi-automatically transferred into the mirror database.
- Prospective period: data will be collected at least once a week by the PI or delegated personnel.

TransFAIR study Dates

TransFAIR Study Start Date (Planned): 15 July 2019
TransFAIR Study End Date (Planned): 30 Nov 2019
Data Base Completed: 07 Dec 2019
Analysis Completed: 15 Jan 2020
Report Completed: 30 Jan 2020

Candidate List of Studies

The list below, is the studies that meet the eligibility criteria:

- Must be conducted at least one of the EHR2EDC partner hospitals. Preference will be given to studies planned at least two of the partner hospitals.
- Must have more than 4 patient's visits in the first six months.
- Should collect local laboratory data, demographics, vital signs

Ammour N, *et al. BMJ Health Care Inform* 2023; 30:e100602. doi: 10.1136/bmjhci-2022-100602

Table 1: List of Candidate reference Clinical Trials

Sponsor	Study No	Phase	Indication	Status (ongoing/ planned)	Local lab (high/medium/low)	Vital signs (high/medium/lo w)	Sites
AstraZeneca	AZ D19BC	3	Cancer	Ongoing	medium	high	IRST, Meldola, Italy
AstraZeneca	AZ D169CC	3b	Cardio	Ongoing			Hospital 12 de Octubre, Madrid, Spain
Janssen	BLC3003	3	Cancer	ongoing	low	low	IRST, Meldola, Italy
Janssen	PCR3011	3	Cancer	preparation/planned	low	low	Hospital 12 de Octubre, Madrid, Spain
Sanofi	TED14856	1b	Cancer	ongoing	high	low	Hospital 12 de Octubre, Madrid, Spain
Sanofi	EFC15156	3a	Diabetes/cardio	ongoing	high	low	AP-HP - Bichat, Paris, France

Ammour N, *et al. BMJ Health Care Inform* 2023; 30:e100602. doi: 10.1136/bmjhci-2022-100602

Method of evaluation

The analysis will compare the same set of data collected in the Clinical Trial (Control DATA) and entered manually in the sponsor eCRF with the same data collected in the TransFAIR study (TransFAIR DATA) using the EHR2EDC module for each pair of studies.
The count of data collected by both methods will be compared to determine the percentage of correct data, achieved by the EHR2EDC transfer.

Each of the data correctly transferred by EHR2EDC, will be qualified by an OK or NOK

- OK: data correctly transferred, which is identical to that collected manually
- NOK: data exposed by the EHR2EDC module, and not validated by the coordinator site

For each study a results file will be produced which will include the following data (*not finalized*)

- Study
- Site
- Patient participation number
- Visit
- Form
- Fields
- Field value / data point TransFAIR
- Value of the field CONTROL (initial, monito) ⍰ only if data collected automatically?
- Queries CONTROL possible (I think it will be necessary to define a game of querie relevant to compare because quite a lot of it will not make sense)

Statistical Analysis

Three levels of analysis will be carried out. A multi-protocol analysis will be conducted, combining the different data files, at each hospital level, then across hospitals.

First level: for a pair of study, comparing the control DATA to the TransFAIR DATA.

Second level: at hospital level, the results of the pair study comparison will pooled for all protocols for a given hospital.

Third level: at the project level, the results of hospital level analysis will be pooled across hospitals.

Analysis Plan - General aspects

- Descriptive statistics will be based on means (+/- standard deviation) or medians [minimum-maximum] depending on the distribution of quantitative variables. The qualitative variables will be described in terms of size and percentage.
- Univariate comparisons will use the usual statistical tests after verification of the distribution of the variables (Chi2 or Fisher's test, t test, anova or their non-parametric equivalents Wilcoxon and Kruskal-Wallis tests).
- The tests and descriptive analyzes will be carried out with a degree of significance of 5%, using the statistical software R and SAS. 95% confidence intervals will be provided for each estimate.

Primary judgment criterion
- An estimate of the proportion with its 95% confidence interval will be provided. The exact calculation method will be used if the approximation of the normal law is not possible

Explanatory analyzes

A subgroup analysis is planned on the following variables:

- Study site
- Type of protocol (trial vs. other type of study)
- Medical specialty concerned by the protocol
- CDISC domain?
- Data type
- Variable (in CDISC terms)

References

El Fadly A, Rance B, Lucas N, Mead C, Chatellier G, Lastic PY, Jaulent MC, Daniel C. Integrating clinical research with the Healthcare Enterprise: from the RE-USE project to the EHR4CR platform. J Biomed Inform. 2011 Dec;44 Suppl 1:S94-102.

Laura Lovett; Pfizer, Ochsner Health team up for clinical trial innovation; https://www.mobihealthnews.com/ February 20, 2019

Nordo A, Levaux H, Becnel L, Jose Galvez, Rao P, Stem K, Prakash E, Kush R; Use of EHRs data for clinical research: Historical progress and current applications - Learning Health System, 16 January 2019

FDA Guidance for Industry - Use of Electronic Health Record Data in Clinical Investigations - July 2018

https://www.fda.gov/drugs/developmentapprovalprocess/formssubmissionrequirements/electronicsubmissions/ucm464653.htm

https://ec.europa.eu/digital-single-market/en/exchange-electronic-health-records-across-eu

https://ec.europa.eu/digital-single-market/en/news/digitalhealtheurope-get-support-your-digital-transformation-health-and-care-activities
